# Comparison of a Real-Time Multiplex PCR and Sequetyping Assay for Pneumococcal Serotyping

**DOI:** 10.1371/journal.pone.0137349

**Published:** 2015-09-03

**Authors:** Felix S. Dube, Suzan P. van Mens, Lourens Robberts, Nicole Wolter, Paul Nicol, Joseph Mafofo, Samantha Africa, Heather J. Zar, Mark P. Nicol

**Affiliations:** 1 Division of Medical Microbiology, Faculty of Health Sciences, University of Cape Town, Cape Town, South Africa; 2 Department of Medical Microbiology and Immunology, St Antonius Hospital, Nieuwegein, the Netherlands; 3 National Health Laboratory Service, Groote Schuur Hospital, Cape Town, South Africa; 4 Department of Paediatrics and Child Health, Red Cross War Memorial Children’s Hospital, Cape Town, South Africa; 5 MRC Unit on Child and Adolesscent Health, University of Cape Town, Cape Town, South Africa; 6 Centre for Respiratory Diseases and Meningitis (CRDM), National Institute for Communicable Diseases of the National Health Laboratory Service, Johannesburg, South Africa; 7 School of Pathology, Faculty of Health Sciences, University of the Witswatersrand, Johannesburg, South Africa; 8 The State Agricultural Biotechnology Centre, Murdoch University, Murdoch, Australia; 9 Centre for Proteomic and Genomic Research (CPGR), Cape Town, South Africa; University of Malaya, MALAYSIA

## Abstract

**Background:**

Pneumococcal serotype identification is essential to monitor pneumococcal vaccine effectiveness and serotype replacement. Serotyping by conventional serological methods are costly, labour-intensive, and require significant technical expertise. We compared two different molecular methods to serotype pneumococci isolated from the nasopharynx of South African infants participating in a birth cohort study, the Drakenstein Child Health Study, in an area with high 13-valent pneumococcal conjugate vaccine (PCV13) coverage.

**Methods:**

A real-time multiplex PCR (rmPCR) assay detecting 21 different serotypes/-groups and a sequetyping assay, based on the sequence of the *wzh* gene within the pneumococcal capsular locus, were compared. Forty pneumococcal control isolates, with serotypes determined by the Quellung reaction, were tested. In addition, 135 pneumococcal isolates obtained from the nasopharynx of healthy children were tested by both serotyping assays and confirmed by Quellung testing. Discordant results were further investigated by whole genome sequencing of four isolates.

**Results:**

Of the 40 control isolates tested, 25 had a serotype covered by the rmPCR assay. These were all correctly serotyped/-grouped. Sequetyping PCR failed in 7/40 (18%) isolates. For the remaining isolates, sequetyping assigned the correct serotype/-group to 29/33 (88%) control isolates. Of the 132/135 (98%) nasopharyngeal pneumococcal isolates that could be typed, 69/132 (52%) and 112/132 (85%) were assigned the correct serotype/-group by rmPCR and sequetyping respectively. The serotypes of 63/132 (48%) isolates were not included in the rmPCR panel. All except three isolates (serotype 25A and 38) were theoretically amplified and differentiated into the correct serotype/-group with some strains giving ambigous results (serotype 13/20, 17F/33C, and 11A/D/1818F). Of the pneumococcal serotypes detected in this study, 69/91 (76%) were not included in the current PCV13. The most frequently identified serotypes were 11A, 13, 15B/15C, 16F and 10A.

**Conclusion:**

The rmPCR assay performed well for the 21 serotypes/-groups included in the assay. However, in our study setting, a large proportion of serotypes were not detected by rmPCR. The sequetyping assay performed well, but did misassign specific serotypes. It may be useful for regions where vaccine serotypes are less common, however confirmatory testing is advisable.

## Introduction

The pneumococcus (*Streptococcus pneumoniae*) is a common cause of invasive disease and respiratory tract infections including bloodstream infections, meningitis, pneumonia and otitis media [1–3]. Patients at risk include those at the extremes of age and the immunocompromised, particularly those affected by cell-mediated immune deficiencies. Colonisation of the nasopharynx with a homologous strain of pneumococci precedes the development of invasive and respiratory tract disease [2,4,5]. Serotyping of the pneumococcal polysaccharide capsule, the immunogenic component of current vaccines, remains the cornerstone of strain characterization. To date, more than 90 capsular serotypes have been described and new ones continue to be described [6,7]. Multiple pneumococcal serotypes can colonize the nasopharynx successively over long period of time, or at any one time [8–10]. Invasive disease is commonly regarded as resulting from a single serotype. Public health programs employ serotype prevalence data from invasive disease to assist vaccine selection. Regular surveillance is required, and relies mostly on phenotypic serotyping methods, most notably the Quellung method developed in 1902 [11]. The antiserum utilised in this assay is costly, methods employed are labour intensive, and require significant technical expertise and experience.

More practical, higher throughput typing techniques are required for expanding public health laboratory services in many areas of the world to support growing disease control programs and epidemiological surveillance. Emerging technologies include alternative culture-based phenotypic methods such as latex agglutination, dot blot ELISA and microbead assays [10,12,13]. While the newer phenotypic methods all have their distinct benefits and often surpass Quellung in terms of rapidity and cost, some of the methods require sophisticated and expensive instruments.

Promising genotypic typing methods that target serotype-specific regions of the *cps* genes have been developed including multiplex Polymerase Chain Reaction (PCR) with subsequent agarose gel electrophoresis [14–16]; restriction fragment length polymorphism (PCR-RFLP) [17]; automated fluorescent capillary electrophoresis (FAF-mPCR) [18]; electrospray ionization mass spectrometry (PCR/ESI-MS) [19]; reverse line blot hybridization assay (mPCR/RLB) [20] and real-time multiplex PCR (rmPCR) [21] including the recently described nanofluidic rmPCR [22]. PCR with subsequent target detection is prone to amplicon contamination and is more labour intensive than rmPCR. rmPCR obviates the need for amplicon manipulation, is highly sensitive, fast and less labour intensive. PCR assays do not require viable isolates and have the potential to detect multiple serotypes simultaneously [21,23–26]. More recently sequetyping, a sequence-based typing method, has been described [27]. There are currently no published head-to-head comparisons of the accuracy of the sequetyping vs. multiplex PCR approaches. Given the heterogeneity and recombinogenic nature of pneumococci, capsular typing tools which infer type from DNA sequence, including target enrichment-based next generation sequencing (NGS) and whole genome sequencing (WGS) [28] are attractive newer methods to complement the molecular typing methods discussed above and may also aid in resolving discrepant phenotypic and genotypic findings.

## Materials and Methods

### Assay validation

**Fig 1:**
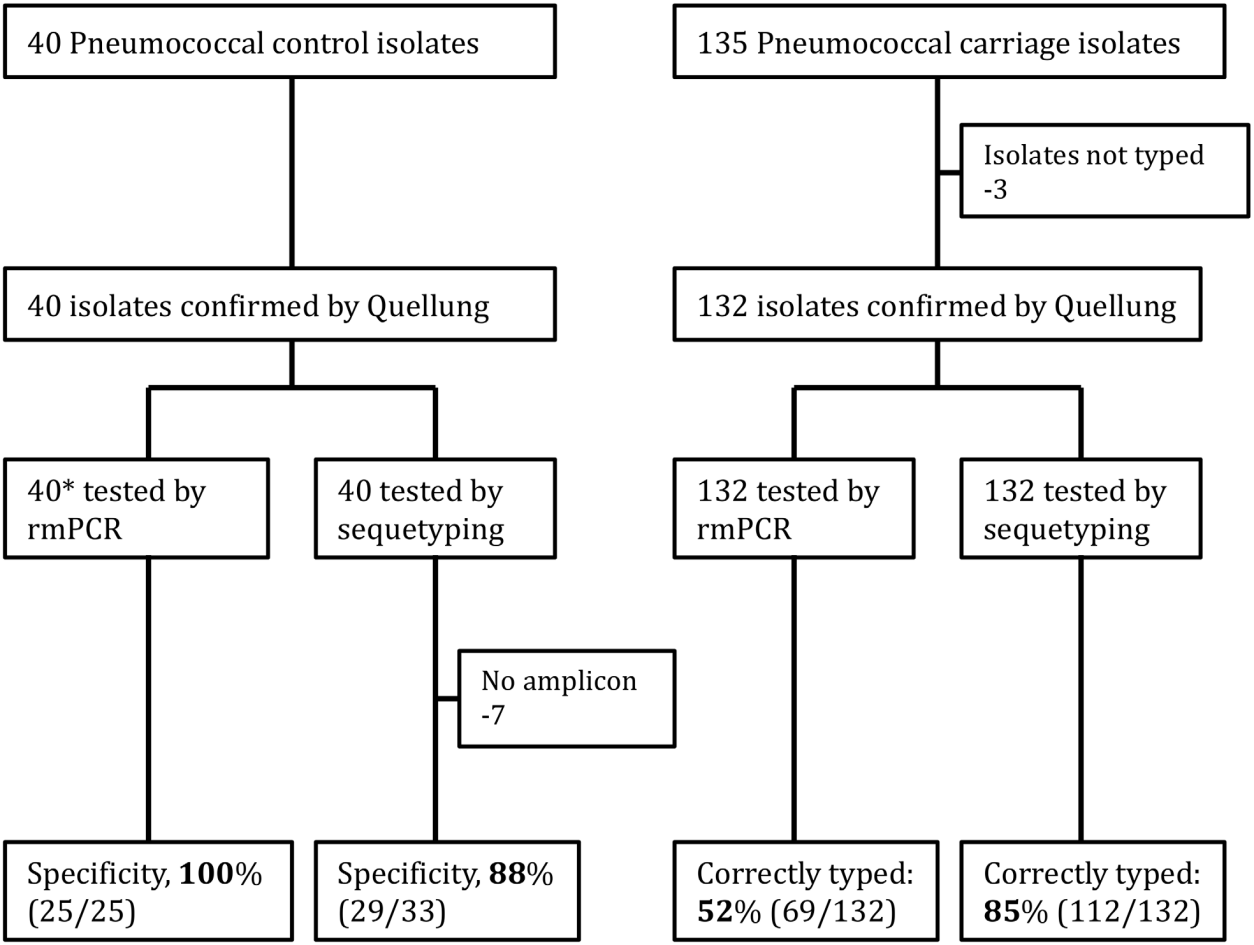
Flow chart showing the pneumococcal isolates included in the study. *Of the 40 isolates that were tested by rmPCR, only 25 were included as part of the rmPCR targets.

Isolates comprised 40 Quellung-typed control strains, Fig 1, (kindly donated by Dr. Anne von Gottberg, Centre for Respiratory Diseases and Meningitis (CRDM), National Institute for Communicable Diseases (NICD), South Africa [29]). These isolates were transported on Dorset egg medium [30], subcultured onto Columbia blood agar base with 2% agar, 5% horse blood and 4 μg/mL gentamicin media (CAG) upon receipt (Green point Media Laboratory of the National Health Laboratory Service, Cape Town, South Africa) and incubated at 37°C in 5% CO_2_ overnight. The resulting colonies were inoculated into in 1 ml skim milk-tryptone-glucose-glycerol (STGG) transport medium frozen at -80°C for batch processing.

Subsequently, 135 pneumococcal isolates (Fig 1) were cultured from nasopharyngeal (NP) swabs that were collected from 83 healthy infants by employing nylon flocked swabs (Copan Italia, Brescia, Italy). Infants were recruited between May 2012 and September 2013 as part the Drakenstein Child Health Study (DCHS), a South African birth cohort study [31]. NP swabs were collected employing the World Health Organization protocol for pneumococcal carriage studies. Briefly, the collected NP swabs were immediately placed into 1 ml STGG, transported on ice to the laboratory and frozen at -80°C for batch processing. After thawing, STGG samples were vortexed for 15 s before a 10 μl aliquot was inoculated onto Columbia blood agar base with 2% agar, 5% horse blood (BA) plates and incubated at 37°C in 5% CO_2_ overnight. Presumptive pneumococcal isolates were identified by colony morphology, α-hemolysis and ethylhydrocupreine (optochin) disk susceptibility (Oxoid, Basingstoke, UK) as previously described [32–34].

### Nucleic acid extraction

Prior to rmPCR and sequetyping, all isolates were subjected to nucleic acid extraction employing a heat lysis method as previously described [35]. Briefly, a sweep of pneumococcal colonies was obtained from primary BA plates that were inoculated with thawed STGG aliquots containing either pneumococcal control strains or carriage isolates. The colony sweeps were resuspended in 100 μl of phosphate-buffered saline, pH 7.4 (PBS; Sigma-Aldrich, St. Louis, MI) thereafter heated at 95°C for 5 min. The supernatant containing genomic DNA (gDNA) was ten-fold serially diluted in PBS before nucleic acid amplification.

### Real-time multiplex PCR

The rmPCR, designed as a 7x3-plex that targets 21 serotypes. We used the multiplex scheme for an African region, based on a relatively limited sample observed [21]. This assay targets all the pneumococcal serotypes included in PCV13 and 8 other additional serotypes/-groups (PCV13 serotypes: 1, 3, 4, 5, 6A/6B, 7F/7A, 9V/9A, 14, 18C/18A/18B/18C, 19A, 19F, 23F, 23A; The 8 additional serotypes/-groups include: 2, 6C/6D, 11A/11D, 12B/2F/46, 15A/15F, 16F, 22F, 33A/33F). Briefly, the PCR reaction comprised 12.5 μl of 2X SensiFAST Probe No-ROX One-Step master mix (Bioline, Taunton, MA), primers and probes for serotype as described by Pimenta et al, 5 μl gDNA (diluted 1:1000) and nuclease/RNase-free water (Applied Biosystems, Irving, CA) for a final reaction volume of 25 μl (S1 Table). The thermal cycling conditions consisted of initial denaturation at 95°C for 10 min, followed by 40 cycles of denaturation at 95°C for 15 s and annealing/extension at 60°C for 1 min employing a CFX96 Touch Real-Time PCR amplification system (Bio-Rad Laboratories, Hercules, CA)

### Sequetyping

The assay was performed as previously described [27] with minor modification: the PCR reaction comprised 12.5 μl of 2X KAPA T*aq* Ready Mix (KAPA Biosystems, Boston, MA), 1 μl of primer mix, 2 μl gDNA (diluted 1:10), 8.5 μl nuclease/RNase-free H_2_O (Applied Biosystems) in a final volume of 25 μl (S2 Table). Thermal cycling consisted of an initial denaturation at 95°C for 5 min, followed by 30 cycles of denaturation at 95°C for 30 s, annealing at 65°C for 30 s, and extension at 72°C for 90 s employing an Applied Biosystems 2720 Thermal Cycler (Applied Biosystems). The PCR products were separated by electrophoresis in 1.5% agarose gel (SeaKem LE Agarose; Lonza, Rockland, ME) for 45 min at 80 V in a 1X Tris-acetate EDTA buffer. Ethidium bromide-stained DNA products were visualized under UV illumination and sized by using a 1–kb DNA molecular size marker (HyperLadderv1-kb; Bioline).

PCR products were prepared for sequencing employing Exo-SAP IT (Affymetrix, Maumee, OH) according to the manufacturer’s instructions. Prepared amplicons were submitted for cycle sequencing employing the BigDye Sequence Terminator kit V3.1 (Applied Biosystems) and analysed on an ABI 3500 XL Genetic Analyzer (Appplied Biosystems) by Inqaba Biotech (Inqaba Biotechnical Industries [Pty] Ltd, Pretoria, South Africa). Sequencing was performed in both directions using forward (*cps*1), 5’-GCA ATG CCA GAC AGT AAC CTC TAT-3’, and reverse (*cps*2), 5’-CCT GCC TGC AAG TCT TGA TT-3’ primers.

DNA sequences obtained were assembled and edited using DNA Baser Sequence Assembler v4 (www.DnaBaser.com). The consensus sequences were used to interrogate the GenBank database (http://www.ncbi.nlm.nih.gov/blast/) and assign a serotype using the criteria as per protocol [27]. Briefly, the serotype of the *wzh* nucleotide sequence from GenBank with the highest BLAST bit score was assigned, provided that sequence identity was >98% with the query amplicon nucleotide sequence. To automate the above process, a Java-based program, Sequetyper (available at http://www.gematics.com/sequetyper.html) was developed and validated to automatically analyse and determine the pneumococcal serotype based on interrogation of GenBank with the input forward and reverse sequences of the generated *wzh* amplicon. This application is suitable for high-throughput analysis of sequetyping data (S1 Fig).

### Quellung testing

Pneumococcal control and nasopharyngeal isolates were submitted to CRDM for quellung testing using specific anti-sera (Statens Serum Institut, Copenhagen, Denmark). Serotype 6C was distinguished from serotype 6A by PCR [36], while serotype 25A and 38 were undistinguishable and hence reported as 25A/38 [37].

### Next-generation sequencing

Three pneumococcal carriage isolates serotyped as 16F by Quellung and rmPCR but identified as 9V by sequetyping were subjected to WGS. The 3 discordant isolates as well as a control strain identified as 9V by Quellung, sequetyping and rmPCR were also included. Briefly, gDNA was isolated with a Wizard Genomic DNA Purification Kit (Promega Corporation, Fitchburg, WI) according to the manufacturer's instructions. The gDNA quality was assessed using the Qubit Fluorometer (Life Technologies, Carlsbad, CA), the NanoDrop ND-1000 (Life Technologies) and agarose gel electrophoresis used to determine absolute concentration, polyphenolic/polysaccharide/chaotropic salt contamination and gDNA integrity respectively. Quantified gDNA was submitted to the Centre for Proteomics and Genomic Research (CPGR) for WGS. Briefly, sequencing libraries were generated using the Nextera XT DNA Sample Prep Kit (Illumina, San Diego, CA) and the libraries were indexed according to the dual-bar cording protocol (with i7 and i5 primers) using the Nextera XT Index Kit (Illumina). Libraries were then normalized, pooled, and a 5% PhiX control added before sequencing with the Illumina MiSeq Reagent Kit v2 (500 cycle) on the Illumina MiSeq system.

### De novo sequence assembly

The quality of the output sequence data was assessed using FastQC [38] and sequencing adapters were trimmed using Trimmomatic [39]. The 3'-end nucleotides with PHRED scores below 20 were trimmed using the fastx_trimmer tool of FASTX toolkit (http://hannonlab.cshl.edu/fastx_toolkit) [40]. The sequence data was then assembled *de novo* using SPAdes v3.0.0 assembler [41]. Draft genome assemblies were annotated individually using RAST (Rapid Annotation using Subsystem Technology) [42]. The contigs containing putative *cps* regions were identified through the standalone *blastall* homology searches against the 16F (Accession: CR931668) and 9v (Accession: CR931648) annotated reference genomes and then extracted to a separate file using a shell command based on SAMtools [43]. These contigs were then aligned and visual representation of the alignments was performed using the Artemis Comparison Tool (ACT) v6 and WebACT [44].

### Data analysis

Results of the two molecular serotyping assays up to the serogroup level were compared with serotyping results obtained by Quellung testing. In cases of discordance between the two molecular serotyping assays, the results were confirmed by Quellung testing. Serotype distribution was determined based on Quellung results. Where more than one isolate was tested from the same child, isolates of the same serotype were included only once in the analysis.

### Ethical consideration

Ethical approval was obtained from the Human Research Ethics Committee of the Faculty of Health Sciences, University of Cape Town (HREC ref: 062/2011) and the Western Cape Provincial Child Health Research committee. Mothers provided written informed consent at enrolment.

## Results

### Real-time multiplex PCR

Of the 40 pneumococcal control isolates subjected to rmPCR, 25 isolates yielded a positive signal; 15 isolates failed to yield detectable amplification signal. Of the 25 rmPCR positive isolates, results were all (25/25) concordant with Quellung confirmed serotypes (Table 1).

**Table 1:** Concordance of molecular serotyping results of pneumococcal control strains.

Serotype	rmPCR^a^	Sequetyping
1	1	1
2	2	No ID^b^
3	3	3
4	4	4
5	5	5
6A	6A/6B	6A
6C	6C/6D	6C/6D
7F	7F/7A	7F/7A
9V	9V/9A	9V
11A	11A	Neg^c^
12B	12F/12A/12B/44/46	12B
12F	12F/12A/12B/44/46	Neg
14	14	14
15A	15A/15F	15A
15F	15A/15F	15F
16F	16F	9V^d^
18C	18C/18A/18B/18F	18B^d^
19A	19A	Neg
19F	19F	19F
22F	22F/22A	Neg
23A	23A	23A
23F	23F	Neg
33A	33F/33A/37	33A/33F/35A
33F	33F/33A/37	33A/33F/35A
46	12F/12A/12B/44/46	12A
7C	N/A*	7C
8	N/A*	8
9N	N/A*	9N
10A	N/A*	10A
10F	N/A*	10C/10F
11B	N/A*	Neg
15B	N/A*	15B/15C
21	N/A*	Neg
23B	N/A*	23B
24B	N/A*	24B
27	N/A*	27
28A	N/A*	28A
31	N/A*	31
35B	N/A*	35B/35C
35C	N/A*	33A/33F/35C

^a^rmPCR: real-time Multiplex PCR.

N/A* = serotype not included in the rmPCR panel.

No ID ^b.^ = sequence identity of ≤98% with sequences in GenBank.

Neg^c^ = negative sequetyping PCR result.

^d^The Sequetyping assay mistyped serotype 18C as 18B.

### Sequetyping

Of the 40 pneumococcal control isolates subjected to sequetyping, 33 isolates yielded single amplicons of ~ 1,061 bp; 7 isolates failed to yield detectable amplicons. Sequence analysis yielded 29/33 (88%) sequetype-Quellung concordant results (Table 1). Of the four discordant results, Quellung 16F was identified as 9V by sequetyping, Quellung 46 was identified as 12A by sequetyping while Quellung 18C was identified as 18B. The fourth discordant isolate yielded no match (>98%) when submitted to GenBank and is considered novel (GenBank submission accession number: BankIt1792036). The *wzh* PCR was negative in 7/40 (18%) control isolates tested, which consequently could not be sequetyped. Detailed results are provided in Table 1.

### Carriage isolates

Of 135 pneumococcal isolates tested, 132 (98%) were assigned a serotype/-group by the Quellung reaction (Tables 2 and 3). Three (3) isolates could not be typed by either Quellung or molecular methods. A total of 69 (52%) isolates were assigned a serotype covered by the rmPCR assay. Of these, the rmPCR assay assigned the correct serotype to all 69 isolates (Tables 2 and 3).

**Table 2:** Carriage isolate serotyping method concordance.

Serotype (n)^a^	rmPCR (n) ^b^	Sequetyping (n)	Remarks
1 (1)	100% (1)	100% (1)	
3 (1)	100% (1)	100% (1)	
4 (1)	100% (1)	100% (1)	
6A (2)	100% (2)	50% (1)	2 rmPCR as 6A/6B; 1 sequetyped as 9V
6B (11)	100% (11)	100% (11)	11 rmPCR as 6A/6B
6C (1)	100% (1)	100% (1)	1 rmPCR as 6C/6D; 1 sequetyped as 6C/6D
10A/11A (1)	100% (1)	100% (1)	1 rmPCR as 11A/11D; 1 sequetyped as 10A
11A (8)	100% (8)	75% (6)	8 rmPCR as 11A/11D; 6 sequetyped as 11A/11D/18F; 2 no amplicon in sequetyping
14 (5)	100% (5)	100% (5)	
15A (10)	100% (10)	100% (10)	10 rmPCR as 15A/15F
16F (8)	100% (8)	63% (5)	3 sequetyped as 9V
17F/1^¥^ (2)	100% (2)	100% (2)	2 rmPCR as 1; 2 sequetyped as 1
18C (3)	100% (3)	33% (1)	3 rmPCR as 18A/18B/18C/18F; 1 sequetyped as 18B, 2 sequetyped with low identity score
19A (8)	100% (8)	75% (6)	2 no amplicon in sequetyping
19F (5)	100% (5)	100% (5)	
22F (2)	100% (2)	100% (2)	2 rmPCR as 22A/22F; 2 sequetyped as 22A/22F
7C (1)*	N/A	100% (1)	
9N (4)*	N/A	100% (4)	
10A (8)*	N/A	100% (8)	
13 (14)*	N/A	93% (13)	1 sequetyped as 15B; 13 sequetyped as 13/20
15B (7)*	N/A	100% (7)	7 sequetyped as 15B/15C
15C (7)*	N/A	100% (7)	7 sequetyped as 15B/15C
17F (3)*	N/A	33% (1)	2 sequetyped as 33C
19B (2)*	N/A	100% (2)	1 sequetyped as 19C, 1 sequetyped as 19F
21 (5)*	N/A	100% (5)	
25A/38 (3)* ^c^	N/A	**Neg** ^d^	3 no amplicon in sequetyping
35A (9)*	N/A	89% (8)	8 sequetyped as 33A/33F/35A, 1 sequetyped as 13/20
OMNI NEG (3)	Neg	Neg	
Total (132)	69	112	

^a^The numbers in closed brackets indicate the correct identification of a Quellung-confirmed serotype by the rmPCR and sequetyping assays;

^b^rmPCR: real-time multiplex PCR;

* = serotypes not included in rmPCR assay.

^¥^ Mixed serotypes detected;

Neg^d^ = negative sequetyping PCR result;

^c^Serotype 25A and 38 were undistinguishable by Quellung and hence reported as 25A/38 [37].

**Table 3:** Summary of molecular serotyping results of pneumococcal nasopharyngeal isolates from healthy children compared with the serotype determined by the Quellung reaction.

		rmPCR^a^, correctly serotyped^b^			Sequetyping, correctly serotyped^b^			Total
		Yes	No	Negative^c^	Yes	No	Negative^c^	
Quellung	Typeable	69	0	63^*¥*^	112	13	7	132
	nontypeable	0	0	3	0	0	3	3
Total		69	0	66	112	13	10	135

^a^ rmPCR: real-time multiplex PCR.

^b^ isolates typed correctly to the serogroup level compared with phenotypic Quellung reaction results.

^c^ Negative: no amplification.

^¥^ all serotypes not covered by the rmPCR panel.

Of the 135 pneumococcal nasopharyngeal isolates that were sequetyped, 125 isolates yielded single amplicons of ~ 1,061 bp. A correct serotype/-group was determined in 112 (85%) of the 132 nasopharyngeal isolates. The partial *wzh* sequence of 2/3 Quellung 18C isolates did not match any of the pneumococcal *wzh* sequences in GenBank with >98% identity while the third was determined as 18B. The *wzh* PCR was negative for seven isolates of which three were serotype 25A/38, two were serotype 11A and two were serotype 19A, as confirmed by Quellung testing. Consistent misidentifications by sequetyping, occurring in more than one isolate, were observed for serotype 16F (two isolates sequetyped as 9V) and for serotype 17F (two isolates sequetyped as 33C).

**Fig 2:**
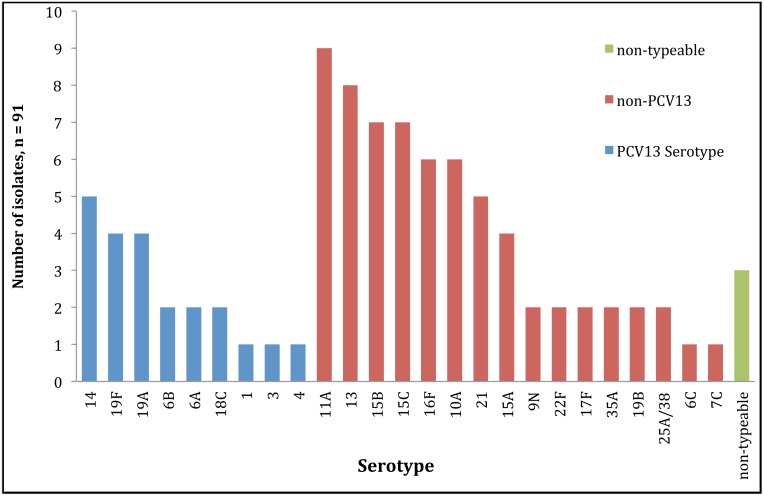
Serotype distribution of nasopharyngeal pneumococcal isolates. The figure includes serotypes detected from the Drakenstein Child Health Study, determined by Quellung reaction, excluding duplicate serotypes from the same infant. Blue = serotypes included in PCV13; Red = serotypes not included in PCV13. Green = non-typable isolates.

Fig 2 shows the serotype distribution of the pneumococcal isolates, excluding duplicate isolates of the same serotype from the same infant. The most frequently identified serotypes were 11A (9 infants), 13 (8 infants), 15B, 15C (both 7 infants), 16F and 10A (both 6 infants). Of the 91 isolates (the total number of isolates when calculating each serotype only once per child), 22 (24%) were serotypes included in PCV13 while 69 (76%) serotypes were not.

### Next-generation sequencing

A total of 14.3 million paired-end sequence reads (2 x 250) were obtained for the four samples as shown in Table 4. The quality control steps used preserved the sequence number though reducing the sequence read length to 230 forward, and 120 reverse (230–120 fr) respectively.

**Table 4:** NGS data and assembly metrics.

Isolate ID	Paired sequence reads	Number of contigs	N50 (Kb)	Draft genome size (Mb)	Sequencing coverage
9v	1 688 340	52	57.1	2.1	144x
16f	4 621 214	47	68.1	2.1	368x
103347	2 226 252	62	77.3	2.1	184x
103385	5 773 884	56	82.3	2.1	437x

**Fig 3:**
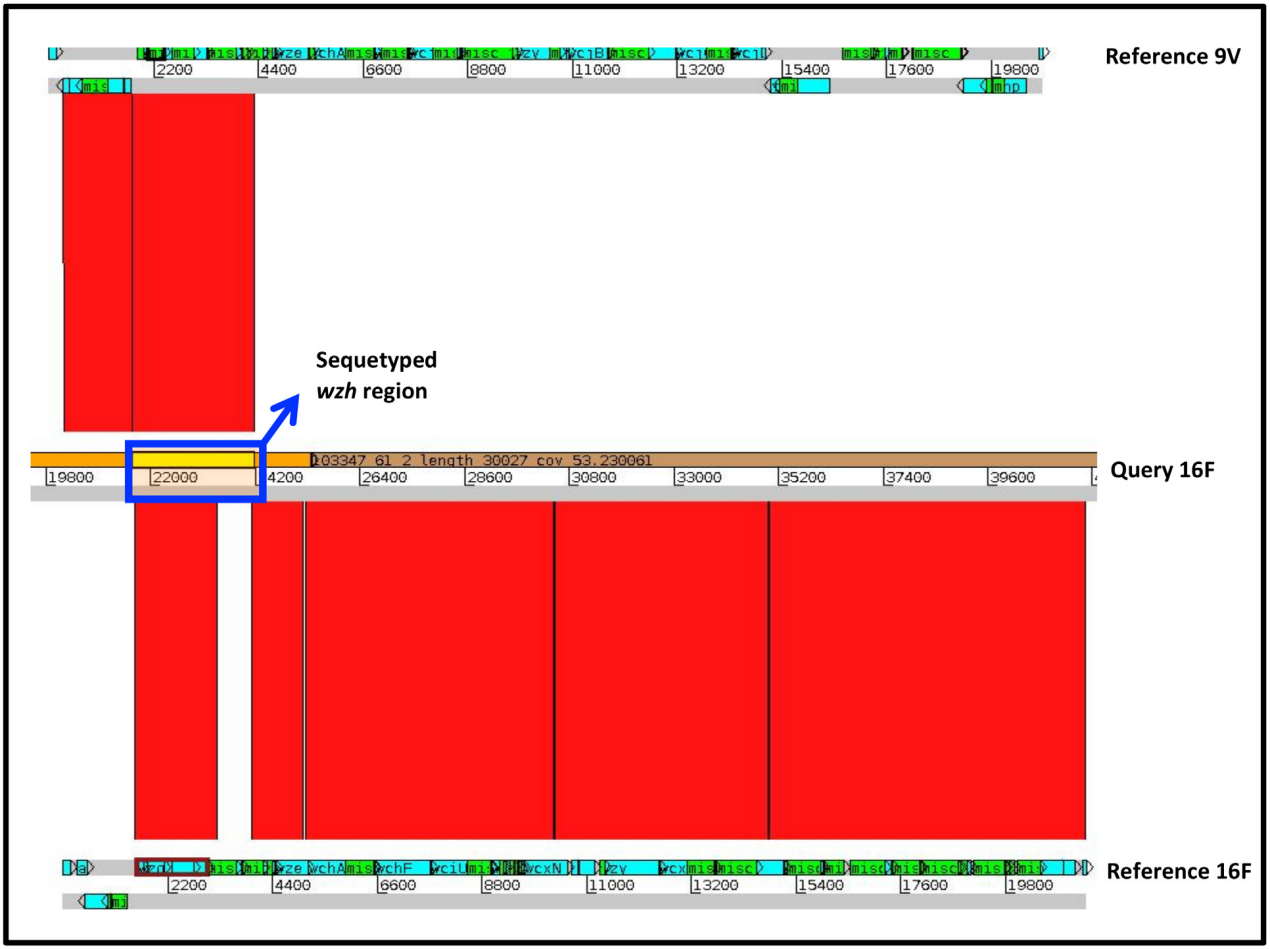
Similarity of 16F-like capsular polysaccharide (*cps*) gene loci. Sequences from pneumococci serotyped as 16F Quellung but sequetyped as 9V was compared to reference 9V (CR931648) and 16F (CR931668) *cps* sequences. Artemis Comparison Tool (ACT) was used to generate and view gene homology. The top lines represent the forward and reverse strand of a serotype 9v reference, the middle lines represent the queried 16F strain and the bottom lines shows the 16F reference. The portion of the *wzh* gene that is amplified by the sequetyping assay is shown by the blue rectangle. The clear blocks below the blue box shows regions were the genes that are not similar. BLASTN matches are shown as red bands between sequences, indicating the degree of similarity between the sequences.

**Fig 4:**
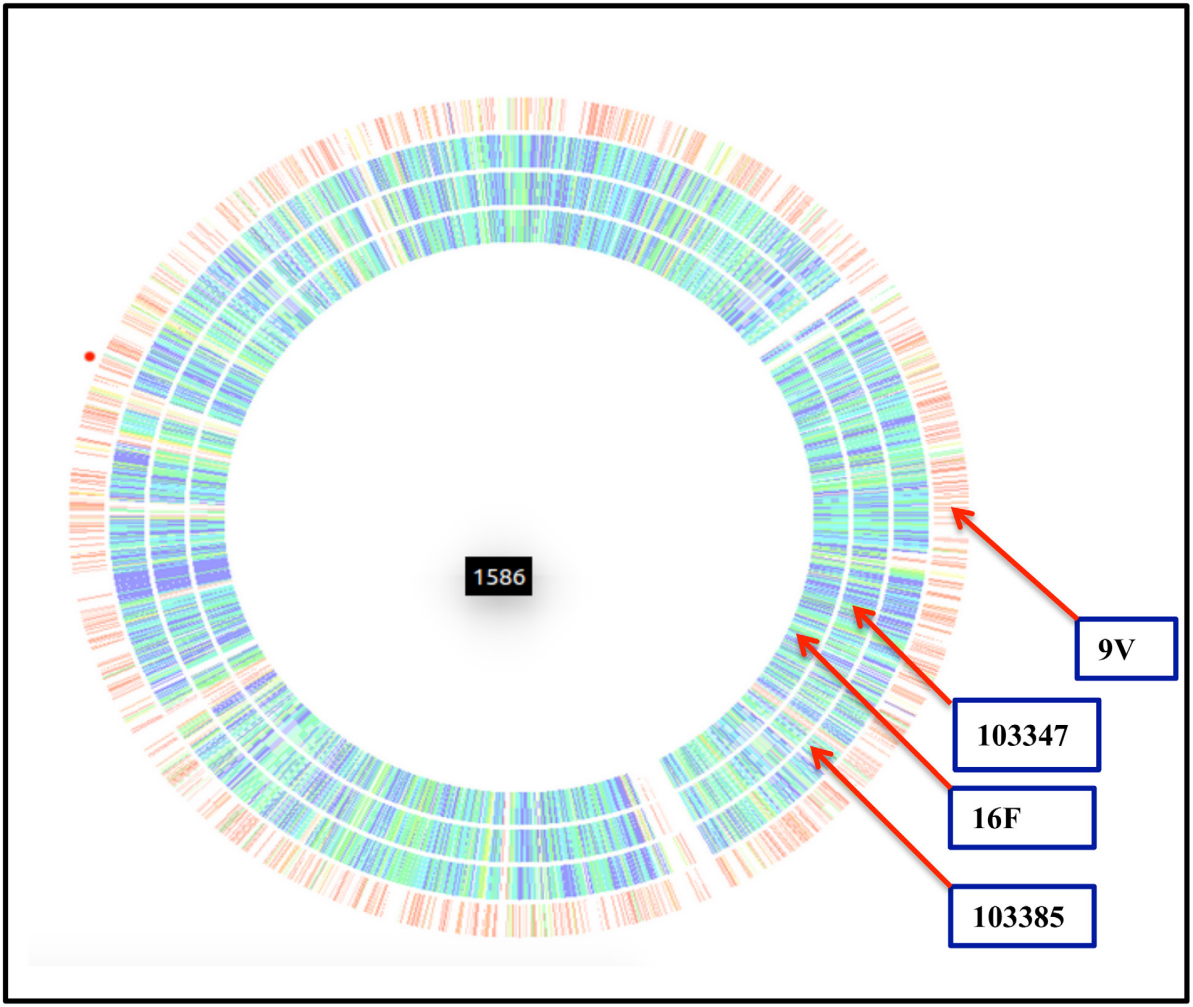
Comparative genome analysis of pneumococcal serotypes 16F and 9V genetic background. When the sequence identities of all four genomes were compared using RAST(Rapid Annotation using Subsystem Technology), the genome backbone of all three 16F (103347 and 103385 from this study and a 16F control strain) were mostly identical but divergent from 9V. The colour codes represent how close or divergent the genomes are. Therefore, similar genome backgrounds will have similar colours.

One of pneumococcal control strains and two DCHS strains were serotyped as 16F in Quellung, but mistyped as 9V by sequetyping. A comparison of the *cps* gene loci showed that the *wzh* sequence of all the three queried 16F strains was entirely 9V-like (Fig 3). This is in contrast to the rest of their *cps* loci: which in terms of structural gene organization as well as specific sequence of these genes were entirely 16F-like. Comparative genome analysis of the annotated gene structure showed a marked clustering for the other three queried 16F serotypes and were all significantly different from the 9V reference (Fig 4). MLST loci of these 16F strains showed a shared a 16F-like-MLST-type, except the third strain (103385) which had a unique glutamate dehydrogenase gene (*gdh*) allele.

## Discussion

To identify a rapid high throughput molecular serotyping assay, rmPCR was compared to sequetyping, in the first place using a panel of 40 control isolates. rmPCR is designed to detect and identify 21 serotypes including all serotypes/-groups in PCV13, all of which were included in our analysis. Concordance with Quellung was 100% (25/25) for those control isolates included in the rmPCR panel. Sequetyping is designed to identify up to 46 different serotypes/-groups, concordance with Quellung for the 40 control strains was 88% (29/33), with failure of sequetyping PCR for 7 strains.

Amongst the pneumococcal carriage isolates tested, the correct serotype/-group could be assigned to 52% (69/132) and 85% (112/132) by rmPCR and sequetyping respectively. Of these, 63/132 isolates were not included in the rmPCR panel. However, the sequetyping assay was theoretically expected to amplify and differentiate all except three isolates (Serotypes 25A and 38) into the correct serotype/-group with some strains giving ambiguous results (serotype 13/20, 17F/33C, and 11A/D/1818F). For those serotypes included in the rmPCR assay, there was good agreement between the results across all three assays. The high number of negative results from rmPCR amongst nasopharyngeal isolates was not surprising since this assay is likely to be less useful in areas where pneumococcal conjugate vaccines have been implemented resulting in serotype replacement which may arise as a result of either serotype unmasking or capsular switching. Data from the United States on invasive pneumococcal isolates showed a decline in serotypes included in the rmPCR assay from 92% (3812/4106) prior to PCV7 implementation to 79% (2939/3708) after PCV7 roll out and a further decrease to 74% (2581/3480) post PCV13 implementation (Unpublished US Active Bacterial Core surveillance data). Amongst our small cohort, non-vaccine serotypes 11A, 13, 15B, 15C, 16F and 10A were the most prevalent serotypes identified. Similarly, data from a number of other post PCV13 surveillance studies have reported serotypes 11A, 15A/B/C, 16, but also 22F, 21 and 34 as prevalent non-vaccine serotypes [45–51]. The original rmPCR protocol [21] did not make reference to any internal control in the assay set up. However, as part of our assay set-up and validation, we screened all the samples with a 16S rRNA PCR to check for inhibition and subjected all rmPCR negative samples to *cps*A PCR to check the integrity of the capsulation locus, although not applicable for serotypes 14, 25, 35A and 38 [52].

The broad range of serotypes that are theoretically detectable by sequetyping is a major advantage of this technique. It is not clear why, in this study, amplification failed for 7/40 (18%) control strains. Interrogation of published gene sequences for these serotypes indicated that these serotypes should generate PCR products with the protocol used here [27]. PCR inhibition was excluded based on successful *lyt*A PCR in all 7 strains. Interestingly, four of the nasopharyngeal cariage isolates that failed to amplify during sequetyping were serotypes for which a similar problem was encountered when sequetyping the control isolates (serotypes 11A and 19A). The remaining three sequetype-negative isolates were of serotype 25A/38 which were expected to be non-amplifiable because of absence of the reverse primer binding site in the *wzd* gene [6,27,53]. Sequetyping misidentified three control strains (for which Quellung and rmPCR were concordant). The sequence obtained from *wzh* amplicon of serotype 2 did not match any of the sequences in GenBank with >98% sequence identity. The original study describing sequetyping did not test this serotype although their *insilico* analysis had predicted that the primer sets should be able to amplify serotype 2 [27]. Misidentification of the serotype 46 isolate as 12A is explained by high relatedness between these serotypes as their *cps* gene clusters are almost identical [6]. Based on our observation of mistyping the 18C PCV13 serotype as 18B by sequetyping, it may be warranted to confirm all 18B results by Quellung. Misidentification of 17F isolates as 33C was predicted by the original sequetyping paper as these serotypes cannot be distinguished based on their *wzh* sequence [27].

We found one control strain and three nasopharyngeal strains that were serotyped as 16F by Quellung, but sequetyped as 9V. Even though the *wzh* sequences of the queried 16F was entirely 9V-like, the serotype specific *wzy/wzx* genes are entirely 16F-like. Based on analysis of the core genome, the 16F control strain was identified as sequence type (ST) 5326, one of the nasopharyngeal isolates was identified as ST4088, while the other nasopharyngeal isolates was a new ST, which was a single-locus variant of ST5326. These sequence types are all commonly associated with serotype 16F [44]. Therefore, our strains seem to be 16F strains in almost every sense, they only have a 9V-*wzh* gene. This structural difference is not expected to have occurred as a result of vaccination, because none of the currently used vaccine formulations include serotype 16F and the exchanged 9V gene does not result in a modified phenotype. In practice, in our setting, each isolate with a 9V sequetype result should be investigated further.

Both molecular assays are able to type many pneumococcal strains only to the serogroup level. Discrimination of individual serotypes within a serogroup may be important for more detailed assessment of carriage and vaccine effectiveness. When selecting a serotyping method, test characteristics other than accuracy may also be relevant. The sequetyping assay, which involves a single amplification step, is inexpensive compared with the rmPCR assay, which is labour intensive, includes many costly PCR probes and is constrained by the limited multiplexing options of real-time PCR. Interpretation of the sequetyping results is based on the publically available GenBank database. An advantage of this is its free accessibility, but the uncontrolled and changing nature of this database could be a risk for the assignment of serotypes. Our automated ‘sequetyper’ application makes analysis of the relevant sequence data for sequetyping rapid and simple. A significant disadvantage of sequetyping is that the targeted *wzh* gene is not serotype-specific and does itself not determine serotype—the results are inferred based on association. It is entirely feasible therefore (as we found here) that for specific serotypes and in particular populations of pneumococci that this association may not correctly predict serotype. The technique is therefore likely only useful for typing pneumococci from populations of pneumococci where such association has been confirmed using another typing technique. In our case this would mean confirming serotype for a smaller subset such as serotype 9V, 13, 20 and serogroup 33. The CDC Streptococcal laboratory has recently provided an update of a conventional multiplex PCR assay (not available at the time of this study) that utilises 41 serotype-specific primer sets to detect upto 70 different pneumococcal serotypes (http://www.cdc.gov/streplab/downloads/pcr-oligonucleotide-primers.pdf). The basis/methodology for this assay is similar to the rmPCR employed here although less costly. The benefits of deducing more than 70 serotypes by this assay needs to be weighed against sensitivity and risk of amplicon contamination.

In conclusion, sequetyping is a useful technique for large scale molecular serotyping of pneumococcal strains, particularly post-PCV introduction, because of the broad range of non-vaccine serotypes that can be detected, low cost and ease of use. Our results suggest the need for an extended and carefully curated database of serotype-specific sequence data, which will increase the accuracy and expand the serotype coverage of the sequetyping method. However, given the potential for gene exchange that could result in false assignment of serotype by sequetyping, it is necessary to confirm serotype assignment using a different method. This may still be cost-saving as it would involve, for example, testing only the specific serotype assigned by serotyping, using the Quellung method, in most instances. The rmPCR assay, ideally extended to include more serotypes is reliable but cost, time required to perform testing, and currently restricted serotype coverage may limit its widespread application for large epidemiological studies. In the future it is likely that WGS will be increasingly used as a tool for serotype inference. WGS has many advantages, in that additional information (such as multi-locus sequence type and antimicrobial resistance) can be inferred from the same dataset without additional testing, and that serotype can be definitively assigned. As sequence costs decline further, bioinformatic pipelines are increasingly automated and the technology is more widely available in low-resource settings it is likely that WGS will replace conventional typing tools for pneumococci.

## Supporting Information

S1 Fig Sequetyper instructions. (PDF)

S1 Table rmPCR setup. (PDF)

S2 Table Sequetyping setup. (PDF)
