# Supplementary material for: Comparison of a Real-Time Multiplex PCR and Sequetyping Assay for Pneumococcal Serotyping
Source: PLoS One. 2015 Sep 3;10(9):e0137349. doi: 10.1371/journal.pone.0137349 (PMC4559314; doi:10.1371/journal.pone.0137349)
Supplement: S1 Fig — (PDF) [file pone.0137349.s001.pdf]

Sequetyper

DNA sequences obtained in section 4.4.1.3 were assembled and edited using DNA Baser Sequence Assembler v4 (www.DnaBaser.com). The consensus sequences were used to interrogate the GenBank database (http://www.ncbi.nlm.nih.gov/blast/) and assign a serotype using the criteria as per protocol [41]. Briefly, the serotype of the *wzh* nucleotide sequence from GenBank with the highest BLAST bit score was assigned, provided that the sequence identity was >98% with the query amplicon nucleotide sequence. When the sequence with the top BLAST bit score was equally shared between two or more serotypes within the same serogroup (i.e., having the same amount of nucleotide sequence variation between query and GenBank database sequences), the result is termed "serogroup-specific." In cases where multiple serotypes of different serotypes/-groups share the highest BLAST bit score, the results were treated as "ambiguous." A misidentified result was one where the top BLAST score corresponds to a sequence of a different serogroup.

The results from this study were used to validate an application called "Sequetyper" which we developed to automatically analyse and determine the pneumococcal serotype based on interrogation of GenBank with the input forward and reverse sequences of the generated *wzh* amplicon. To automate the above process a Java-based program, Sequetyper (available at, http://www.gematics.com/sequetyper.html) was developed. Briefly, Sequetyper will search within a given input directory for the forward and reverse compliment AB1 sequencing trace files using a filenaming convention (detailed in the help menu of the program), perform base base calling using a Phred-scaled quality cutoff value of 0.05. Phred-scaled quality scores are assigned as a logarithmic measure of the base-calling error probalities (Table 4). Phred-scaled quality scores for most sequencing platforms can range from 2 to 63.

For many purposes, a Phred-quaality score of 20 is acceptable. An example is given below in figure 2 where an adenine nucleotide base has been assigned a Phred-scaled quality score of 50. This implies that there is a 1 in 100,000 chance of incorectly calling the adenine nucleotide base. The sequence and quality files are read in and the consensus sequences is used to perform a blastn alignment using a from four stringent criteria. These output files are also generated with the results for review. A typical Phred-quality output report from our study is shown in figure S2. The sequetyper has a local database of nucleotide sequences that are derived from four stringent criteria. The first one is local search within the 92 *cps* sequences made available by the Wellcome Trust [8]; the second criterion returns any pneumococcal *cps* nucleotide sequences; while the third criterion return any pneumococcal nucleotide sequence (This reduces the risk of not picking up any nucleotide sequences that do not indicate the serotype name in its filename, an example of these is given in table S8) excluding shortgun sequences; and the fourth criterion involves a search for pneumococcal nucleotide sequences from the Capsular Sequence typing database [50]. Sequetyper then performs a comprehensive search across all the four criteria, returns a pool of all nucleotide sequences with an identity of >95%. The algorithm then searches within this pool oncly and best alignment with a nucleotide sequence identity of >98% from the four criteria to assign a serotype. The output files are generated in either ".html", ".xls" or ".txt" format. Because the algorithm uses a local BLAST search, the program can run offline and does not require internet connectivity. This application is suitable for high-throughput analysis of sequetyping data. Sequetyper was validated employing 175 paired forward- and reverse sequences generated during the study. Briefly, manually identified sequence identities were re-evaluated blindly through sequetyper and results compared. Two manual vs. sequetyper discordant identities

were obtained, and subsequent to manual re-evaluation assigned according to original sequetyper results. The discordant results was due to as a result of mislabelling of AB1 files such that the algorithm returned with an error while the second was due to a DNABaser miscalling a nucleotide base when generating a contig.

Table 1: Phred quality scores

Phred Quality Score	Error (Probability of incorrect base call)	Base call accuracy (1-Error)
10	1 in 10 = 10%	90%
20	1 in 100 = 1%	99%
30	1 in 1000 = 0.1%	99.9%
40	1 in 10000 = 0.01%	99.99%
50	1 in 100000 = 0.001%	99.999%
60	1 in 100000 = 0.0001%	99.9999%

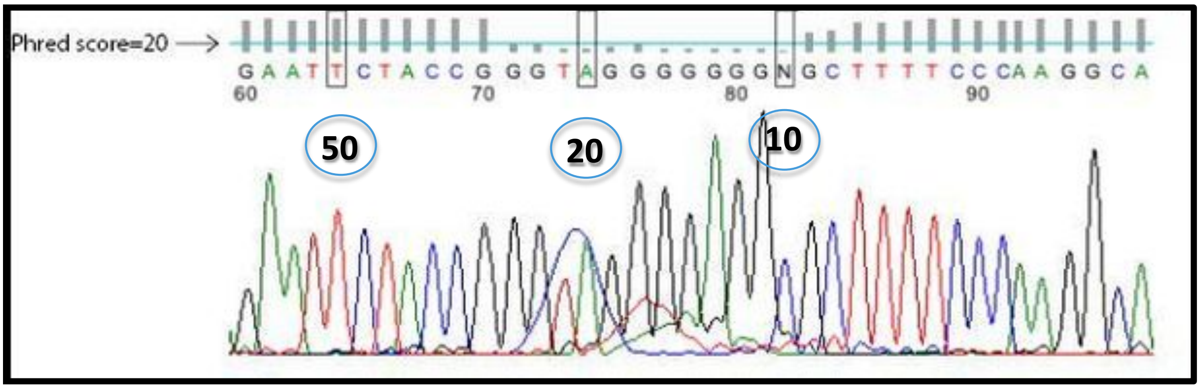

Figure 1: Phred quality scores. The quality of the electropherograph are assesed based on the logarithmic probabilities related to the base-calling error. A high Phred of 50 as in thesse present figure suggests there is one in a 100,000 chances that a Adenine base was called wrong by the sequencing platform.

How to run the Sequetyper program

In order to assign serotypes, download the sequetyper program from http://www.gematics.com/sequetyper.html which is stored as a zip folder. After extracting the program to a specified location:

1) Navigate to the sequetyper folder and and double click the run in order to launch the program for Windows users or use the run.command for MAC operating system users.
2) Click on "File" and then "Open directory"
3) Now navigate to a secified directory that contains the AB1 files which have been labelled using a set convention (See under rebuilding the database for the naming format).
4) Once you have selected a directory, click on the "Open" button
5) A window will open to notify the user if there are any errors in file processing which can include sequences that for below the 0.05 PHRED cutoff or sequences that are duplicated or have a missing compliment sequence.
6) If this QC step is resolved, click on "Proceed anyway"
7) The program will then find all the alignments for the input files.
8) Once the alignment is complete, a report window will open showing the results.

5.3.6.4 Rebuilding the Sequetyper database

In order to rebuild the database of reference sequences the following steps must be carried out:

1) First locate the file called "categoryCatAll.fasta" located in the "db" folder,
2) Open this file for editing in a text editor and
3) Paste in new sequence in fasta format.

Note: The new sequence must have a specific format. The specific format applies to the definition line, the line with the ">" in it. This normally looks something like: *>gi|68643586|emb|CR931683.1|Gene locus A*

It should have the following format:

>w|x|y|z|serotype_A_B_C_2#D

where

- >w|x|y|z|can be anything but
- _A_ is the Pneumococcal serogroup,
- _B _Pneumococcal serotype, which could be designated as F, A, B, C, D, N, L, V
- or left blank for serotypes that do not have any serotypes included within the serogroup,
- _C_is the allele number if applicable and
- _D_ is the Pneumococcal sequence description.

An example of what it should look like is:

>gi|68643586|emb|CR931683.1|serotype_23_a_0_2#Streptococcus pneumoniae strain 1196/45 (serotype 23a) 21,475 bp linear DNA.

The definition line must contain four underscores(_) and one hash(#).

After the sequence has been added, or sequences have been removed, save the file and return to the program. Then

4) Click on 'File' and then 'Rebuild database'

This will rebuild the database using the sequences as they appear in the fasta file. The program will then use the updated information when assigning serotypes.
