# Supplementary material for: Comparison of a Real-Time Multiplex PCR and Sequetyping Assay for Pneumococcal Serotyping
Source: PLoS One. 2015 Sep 3;10(9):e0137349. doi: 10.1371/journal.pone.0137349 (PMC4559314; doi:10.1371/journal.pone.0137349)
Supplement: S1 Table — (PDF) [file pone.0137349.s002.pdf]

Table 1: Primer and probe data for the sequential real-time multiplex PCR assay

GenBank accession no.(*gene*)	Primer/probe ID	Primer/probe sequence (5' -3')	Probe dye	Probe special chemistry	Quencher (3')	nM
CR931632 (*wzy*)	1-F	TTTCATCCCTATGTGTGGTATAG				300
	1-R	GCTTTAGAAGGTAGAGTTAACAAC	FAM	LNA[b]	BHQ1	300
	1-**Probe**	TGCCAAAGCCAGCCAT				100
CR931633 (*wzy*)	2-F	TGTTATCCCATATAAGAACCGAGTGT				300
	2-R	AAAATTACCCCAAAAGCTATCCAA	FAM	"T"[d=] *BHQ1*	BHQ1	300
	2-**Probe**	TTGCAATT"T"CAATTTTTTTGCCCCAATCTC				200
CR931634 (*galU*)	3-F	CCACTAAAGCTTTGGCAAAAGAAA				300
	3-R	CCCGAACGTAAAGCTTCTTCA	HEX	"T"[d=] *BHQ1*	BHQ1	300
	3-**Probe**	TTGTAGACCGCCCCACAA"T"TCATTTTGT				200
CR931635 (*wzy*)	4-F	GCTTCTGCTGTAACTGTTGTGC				300
	4-R	CACCACCATAGTAACCAAAGTTCC	CY5		BHQ2	300
	4-**Probe**	TTCCACAAAAGAAGAGCCTACAGGTAACCCCA				100
CR931637 (wzy)	5-F	CATGATTTATGCCCTCTTGCAA				300
	5-R	GACAGTATAAGAAAAAGCAAGGGCTAA	HEX	"T"[d=] *BHQ1*	BHQ1	300
	5-**Probe**	TCTTCTTCTCA"T"CGTTTCCGCATGCTTTT				200
CR931639 (*wciP*)	6A/6B/6C/6D-F	GTTTGCACTAGAGTATGGGAAGG				200
	6A/6B/6C/6D-R	TAGCCTTTCTGAAAACATTTAGCG	FAM	"T"[d=] *BHQ1*	BHQ1	200
	6A/6B/6C/6D-**Probe**	TGTTCTGCCC"T"GAGCAACTGGTCTTGTATC				200
EF538714 (*wciN*)	6C/6D-F	TTGGGATGATTGGTCGTATTAG				200
	6C/6D-R	CTCTTCAATTAGTTCTTCAGTTCG	FAM	LNA[b]	BHQ1	200
	6C/6D-**Probe**	CCACGCAATTCGCCATC				100
CR931643 (*wzy*)	7F/7A-F	ATGAAGGCTTTGGTTTGACAGG				200
	7F/7A-R	ATTCTCGCCATCAATTGCATATTC	CY5		BHQ2	200
	7F/7A-**Probe**	ACACCACTATAGGCTGTTGAGACTAACGCACA				100
CR931648 (*wzx*)	9V/9A-F	AGGTATCCTATATACTGCTTTAGG				300
	9V/9A-R	CGAATCTGCCAATATCTGAAAG	HEX	LNA[b]	BHQ1	300
	9V/9A-**Probe**	ACACATTGACAACCGCT				100
CR931653 (*wzy*)	11A/11D-F	AAATGGTTTGGATATGGTTTGTTTGG				300
	11A/11D-R	AGTGCTAACTGTAAAACTTGATTATGAG	CY5		BHQ2	300
	11A/11D-**Probe**	ATTCCAACTTCTCCCAATTTCTGCCACGG				100
CR931660 (*wzx*)	12F/12A/12B/44/46-F	GCACCCACGGGTAAATATTCTAC				300
	12F/12A/12B/44/46-R	CAACTAAGAACCAAGGATCCACAG	CY5		BHQ2	300
	12F/12A/12B/44/46-**Probe**	TGCCCACCAACACCAGGTCCAGGT				200

CR931662 (*wzy*)	14-F	AGAGTGTATGAGGAATCC				300
	14-R	ATATATCTACTGTAGAGGGAAT	FAM	"T"[d=] *BHQ1*	BHQ1	300
	14-**Probe**	CGCCAAGTAACA"T"TTCCATTCCATT				100
CR931663 (wzy)	15A/15F-F	AATTGCCTATAAACTCATTGAGATAG				200
	15A/15F-R	CCATAGGAAGGAAATAGTATTTGTTC	FAM	LNA[b]	BHQ1	200
	15A/15F-**Probe**	CCC**G**CA**A**AC**T**CT**G**TCCT				100
CR931668 (*wzy*)	16F-F	TAATGTTATGACCTTGGTAATCTTCCC				300
	16F-R	TCCCAAAGGATAATCAATAACTTTTAGAAG	HEX	"T"[d=] *BHQ1*	BHQ1	300
	16F-**Probe**	AGCCATAAGTCT"T"CCAAATGCTTAACCGCT				100
CR931673 (*wzy*)	18C/18A/18B//18F-F	TCGATGGCTAGAACAGATTTATGG				200
	18C/18A/18B//18F-R	CCATTGTCCCTGTAAGACCATTG	HEX		BHQ1	200
	18C/18A/18B//18F-**Probe**	AGGGAGTTGAATCAACCTATAATTTCGCCCC				100
CR931675 (*wzy*)	19A-F	CGCCTAGTCTAAATACCA				200
	19A-R	GAGGTCAACTATAATAGTAAGAG	FAM		BHQ1	200
	19A-**Probe**	TATCAATGAGCCGATCCGTCACTT				100
CR931678 (*wzy*)	19F-F	TGAGGTTAAGATTGCTGATCG				300
	19F-R	CACGAATGAGAACTCGAATAAAAG	CY5	LNA[b]	BHQ1	300
	19F-**Probe**	CGC**A**CT**G**TC**A**AT**T**CACCTTC				100
CR931682	22F/22A-F	TCTATTAAATAACCCATTGGAATTGAAACG				200
(*wcwV*)	22F/22A-R	TCGCAATTGAAGACCACATAAACTG	HEX	"T"[d=] *BHQ1*	BHQ1	200
	22F/22A-**Probe**	TCCGTAAT"T"CGCTTATGGGCACATTCTCCA				200
CR931683 (*wzy*)	23A-F	CTCCCCTCCATTACCCATTTGG				200
	23A-R	TGAAGAAAGTGCTGTTTGTGAACC	CY5	"T"[d=] *BHQ2*	BHQ2	200
	23A-**Probe**	AGCTAGAAC"T"CCCACACTCCCTACTCCCA				100
CR931685 (*wzy*)	23F-F	GACAGCAACGACAATAGTCATCTC				300
	23F-R	TCCATCCCAACCTAACACACTTC	CY5	"T"[d=] *BHQ2*	BHQ2	300
	23F-**Probe**	ATTGTGTCCA"T"AACCCTTCGTCGTATTTCCAAAG				200
CR931702 (*wzy*)	33F/33A/37-F	GGAACTGGTTCAGCAACTATACG				200
	33F/33A/37-R	GGTTCTAAGACCGTCTGAAATACC	HEX	"T"[d=] *BHQ1*	BHQ1	200
	33F/33A/37-**Probe**	CCCCAAATAGGAC"T"TTTCTGCCATGCCAAA				200

[a]Abbreviations: FAM, 6-carboxyfluorescein; HEX, hexachloro-6-carboxyfluorescein; ROX, 6-carboxy-X-rhodamine; CY5, indodicarbocyanine; BHQ, black hole quencher; ID, identification; LNA, locked nucleic acid.

[b]Locked nucleic acid nucleotides are underlined.

[d] "T" black hole quencher placed internally on the thymidine, highlighted with a green background. Table adopted from Pimenta *et al* [40].
