# Supplementary material for: Comparison of a Real-Time Multiplex PCR and Sequetyping Assay for Pneumococcal Serotyping
Source: PLoS One. 2015 Sep 3;10(9):e0137349. doi: 10.1371/journal.pone.0137349 (PMC4559314; doi:10.1371/journal.pone.0137349)
Supplement: S2 Table — (PDF) [file pone.0137349.s003.pdf]

Sequetyping

Sequetyping was performed according to Leung *et al* [41] with minor modifications: the PCR reaction comprised 12.5 µl 1X KAPA Taq Ready Mix® (KAPA Biosystems, Boston, MA), 1 µl of primer mix (Table 2), 2 µl gDNA (diluted 1:10), 8.5 µl nuclease/RNase-free H_2O (Applied Biosystems) in a final volume of 25 µl. Thermal cycling consisted of an initial denaturation at 95°C for 5 min, followed by 30 cycles of denaturation at 95°C for 30 s, annealing at 65°C for 30 s, and extension at 72°C 90 s employing an Applied Biosystems® 2720 Thermal Cycler (Applied Biosystems). PCR products were separated by electrophoresis in 1.5% agarose gel (SeaKem LE Agarose; Lonza, Rocklnd, ME) for 45 min at 80 V in a 1X Tris-acetate EDTA buffer. Ethidium bromide-stained DNA products were visualized under UV illumination and sized by using a 1-kb DNA molecular size marker (HyperLadder® 1kb; Bioline).

Table 2: Sequetyping assay PCR reaction mix for 80 reactions

Reagent (initial concentration)	1 Reaction (µl)	X80 Reactions (µl)	Final Concentration
2x Mastermix	12.5	1000	1X
Cps Primer_F 10 uM	1	80	400nM
Cps Primer_R 10 uM	1	80	400nM
dH2O	8.5	680	
DNA	2		
total	25	2000	
